# Supplementary material for: Novel Driver Strength Index highlights important cancer genes in TCGA PanCanAtlas patients
Source: PeerJ. 2022 Aug 11;10:e13860. doi: 10.7717/peerj.13860 (PMC9375969; doi:10.7717/peerj.13860)
Supplement: Supplemental Information 2 [file peerj-10-13860-s002.zip › COHORTS/genes plots/age/2021_8_16_14_9_distribution_cohorts_males_55-59.pdf]

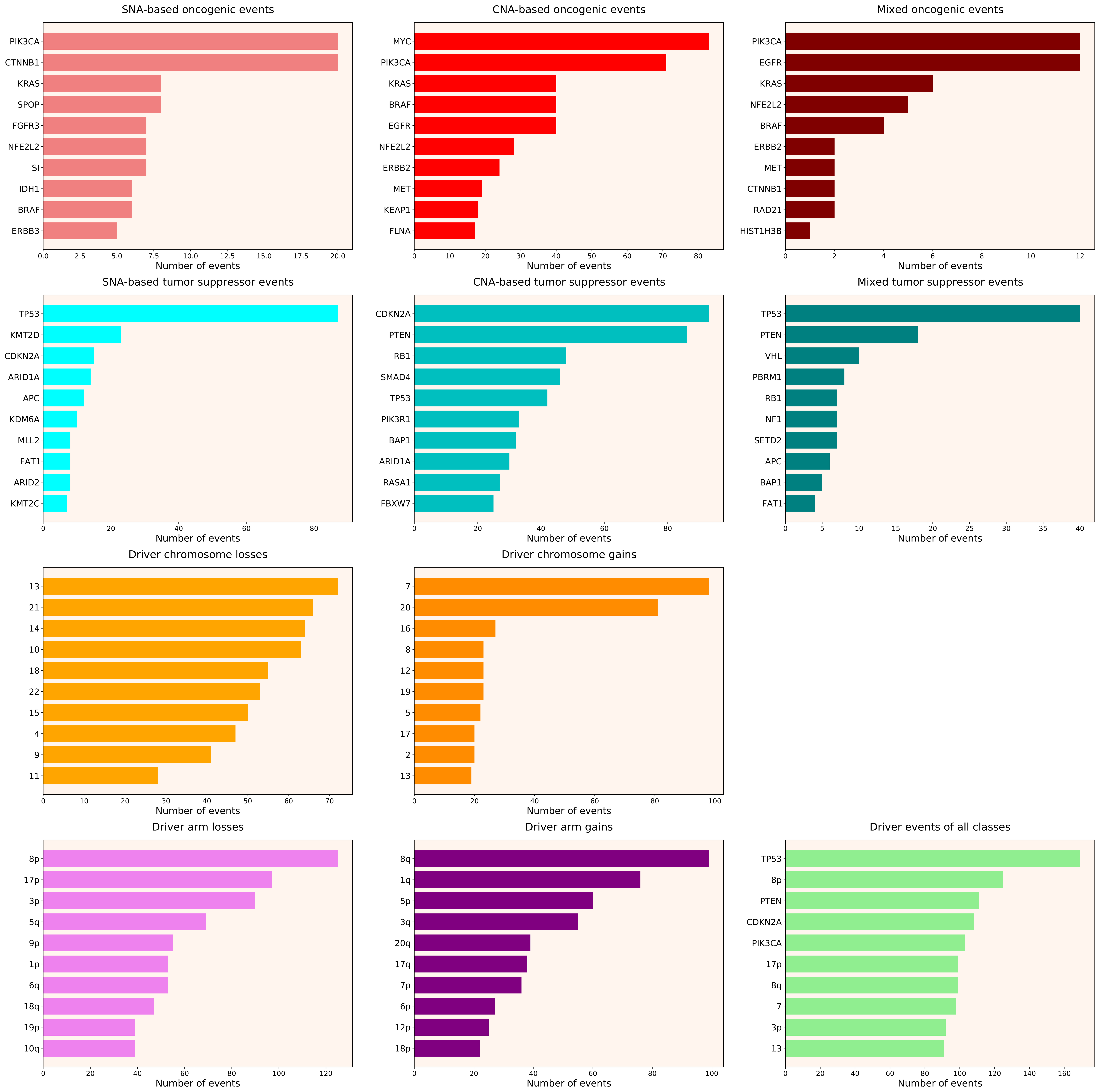
SNA-based oncogenic events
PIK3CA
CTNNB1
KRAS
SPOP
FGFR3
NFE2L2
SI
IDH1
BRAF
ERBB3
0.0 2.5 5.0 7.5 10.0 12.5 15.0 17.5 20.0
Number of events
CNA-based oncogenic events
MYC
PIK3CA
KRAS
BRAF
EGFR
NFE2L2
ERBB2
MET
KEAP1
FLNA
0 10 20 30 40 50 60 70 80
Number of events
Mixed oncogenic events
PIK3CA
EGFR
KRAS
NFE2L2
BRAF
ERBB2
MET
CTNNB1
RAD21
HIST1H3B
0 2 4 6 8 10 12
Number of events
SNA-based tumor suppressor events
TP53
KMT2D
CDKN2A
ARID1A
APC
KDM6A
MLL2
FAT1
ARID2
KMT2C
0 20 40 60 80
Number of events
CNA-based tumor suppressor events
CDKN2A
PTEN
RB1
SMAD4
TP53
PIK3R1
BAP1
ARID1A
RASA1
FBXW7
0 20 40 60 80
Number of events
Mixed tumor suppressor events
TP53
PTEN
VHL
PBRM1
RB1
NF1
SETD2
APC
BAP1
FAT1
0 5 10 15 20 25 30 35 40
Number of events
Driver chromosome losses
13
21
14
10
18
22
15
4
9
11
0 10 20 30 40 50 60 70
Number of events
Driver chromosome gains
7
20
16
8
12
19
5
17
2
13
0 20 40 60 80 100
Number of events
Driver arm losses
8p
17p
3p
5q
9p
1p
6q
18q
19p
10q
0 20 40 60 80 100 120
Number of events
Driver arm gains
8q
1q
5p
3q
20q
17q
7p
6p
12p
18p
0 20 40 60 80 100
Number of events
Driver events of all classes
TP53
8p
PTEN
CDKN2A
PIK3CA
17p
8q
7
3p
13
0 20 40 60 80 100 120 140 160
Number of events
